# Unveiling Oral Cancer Epidemiology in Pakistan: Insights From a Case–Control Study

**DOI:** 10.1155/ghe3/9982580

**Published:** 2025-06-05

**Authors:** Arifa Shabir, Sara Kazmi, Muhammad Usman Rashid, Iqra Mubeen, Arif Jamshed, Syed Raza Hussain, Naila Malkani

**Affiliations:** ^1^Department of Zoology, GC University, Lahore, Pakistan; ^2^Department of Basic Sciences Research, Shaukat Khanum Memorial Cancer Hospital and Research Centre (SKMCH & RC), Lahore, Pakistan; ^3^Department of Clinical and Radiation Oncology, Shaukat Khanum Memorial Cancer Hospital and Research Centre (SKMCH & RC), Lahore, Pakistan; ^4^Department of Surgical Oncology, Shaukat Khanum Memorial Cancer Hospital and Research Centre (SKMCH & RC), Lahore, Pakistan

**Keywords:** epidemiology, oral cancer, Pakistan, prevention, tobacco

## Abstract

**Objectives:** Oral cancer (OC) poses a growing health concern in Pakistan, emerging as the second-most diagnosed cancer in the country. The escalating incidence and mortality rates of OC place considerable strain on the health system. This study aims to delineate the factors contributing to the elevated incidence of OC in Pakistan.

**Methods:** A hospital-based case–control study involving 688 participants above 18 years old was conducted. Participants were evaluated for reported OC risk factors.

**Results:** Findings indicate a heightened susceptibility among men (71.5%) to develop OC in Pakistan. Factors contributing to OC include advancing age (AOR 1.12, 95% CI 1.13–1.18, *p*=0.001), low SES (61.4%), and limited education. Significant risk was associated with chewing tobacco (niswar) AOR 6.83, 95% CI 2.67–17.45, *p*=0.001), areca nut (AOR 4.99, 95% CI 1.51–16.45, *p*=0.001), and pan (AOR 7.90, 95% C1 3.19–19.59, *p*=0.001). Parental consanguinity increased OC incidence (AOR 4.72, 95% CI 1.12–4.14, *p*=0.02). Physical activity had no association with OC (AOR 0.41, 95% CI 0.23–0.75, *p*=0.004). Excessive sunlight exposure appeared to be associated with OC (AOR 0.15; 95% CI: 0.08–0.28, *p*-value: 0.001). At the same time, cigarette smoking and alcohol are not significant factors for the development of OC in Pakistan.

**Conclusion:** The study underscores the elevated prevalence of OC among Pakistani men, attributable in part to lower literacy rates and inadequate access to healthcare facilities. The implementation of targeted prevention strategies informed by these epidemiological insights is essential for mitigating the burden of OC in the region.

## 1. Introduction

Cancer ranks as the second major cause of morbidity and mortality worldwide, with the global cancer burden surging to 20 million cases and 9.7 million deaths in 2022 [1]. Oral cancer (OC) stands as the most common form of neoplasm among head and neck cancers, involving a multistage process of precancerous lesion development, invasion, and metastasis [2]. Globally, OC accounts for approximately 3.8% of all cancer cases, with 389,846 new cases and 188,438 deaths annually [3]. The incidence of OC exhibits a variable geographic distribution, with the highest prevalence in Asian countries, particularly in Southeast Asia [1, 3, 4].

According to the Global Cancer Observatory (GCO), the annual incidence of OC in 2022 reached 389,846 cases worldwide, with the highest number recorded in Asia (258,440), followed by Europe (62,073) and North America (30,992) [1]. While OC has traditionally been identified primarily among males, in many Southeast Asian populations, it is also becoming prevalent among females [5]. In Pakistan, OC has become a significant burden on the healthcare system, being the most prevalent cancer among males (12.9%) and the second-most common among females (9.5%) [3].

OC has a well-established etiology, with tobacco use (both smoking and smokeless forms), betel quid chewing, alcohol consumption, and human papillomavirus (HPV) infection recognized as major risk factors (IARC Monographs, Handbook 19). Additionally, physical inactivity, ultraviolet (UV) radiation exposure, and low socioeconomic status (SES) contribute to OC occurrence and prognosis [6–9]. While dietary habits influence cancer risk, recent evidence suggests that coffee consumption may have protective effects rather than being risk factors. However, there is limited evidence for the association between tea consumption and OC. In low-income and developing countries, a lack of awareness, advanced-stage diagnosis, tumor site, histological grading and treatment type may influence the recurrence rate of OC [10].

This study aims to estimate the distribution of OC in the Pakistani population according to gender, age, patient habits, site of involvement, and histopathological grading. Given that OC is considered preventable through early detection and treatment strategies, the findings of this study will underscore the importance of the widespread implementation of known preventive healthcare measures in Pakistan to enhance early detection of OC and improve disease prognosis.

## 2. Methods

### 2.1. Study Design and Study Population

This study employed a prospective case–control design to investigate the association between different demographic and clinical parameters in OC patients compared to healthy controls. From October 2019 to March 2023, a total of 277 OC patients and 411 healthy controls were recruited from Shaukat Khanum Cancer Memorial Hospital and Research Center, Lahore (SKMCH&RC), and Mayo Hospital (MH), Lahore, Pakistan.

The sample size was calculated using the EpiTool online program (https://epitools.ausvet.com.au), with 95% confidence level and 80% power. A 2:1 case-to-control ratio was employed to enhance statistical power and reduce selection bias. The initial sample size requirement was 561 participants, but after adjusting a 10% dropout rate and missing data, the final sample size was set at 688. A convenient sampling approach was used, ensuring rigorous inclusion and exclusion criteria to enhance the representativeness of both cases and controls while minimizing selection bias.

### 2.2. Ethical Approval and Participant Selection

Ethical approval for this study was obtained from the Scientific Review Board Committee (SRC) and the Institutional Review Board of SKMCH&RC (IRB-19-10), as well as Government College University Lahore, Pakistan (GCU-11B-195).

Only pathologically confirmed OC patients registered at the hospitals were included in the study. Controls were enrolled based on personal and family history free of OC. Participants aged 18 years or above from various regions of Pakistan were selected according to the study's inclusion criteria. Before enrollment, written informed consent was obtained from all participants after a trained study coordinator explained the study objectives. Participants were excluded if they did not meet the inclusion criteria, refused to provide information, or had missing contact details.

### 2.3. Data Collection Tool

Data were collected using structured report forms designed separately for cases and healthy controls. A trained clinical research officer administered the report forms, which were designed in both English and Urdu (Pakistan's national language) to ensure inclusivity. The data collection tool included various social and demographic variables, such as gender, age, ethnicity, smoking status, consumption of niswar, betel quid, paan, alcohol, hot beverages (tea and coffee), oral health indicators (dental implants, gum disease, and mouth ulcers), and medical history (thyroid disease and family history of cancer). For OC patients, clinicopathological data were also documented such as the tumor site, size, recurrence status, histological subtype, TNM staging, and metastasis status. All data underwent double-checking to ensure accuracy and were stored in both hard and digital formats.

### 2.4. Data Analysis

Statistical analysis was performed using SPSS software (version 27.0; SPSS, Chicago, IL, USA). Continuous variables were expressed as mean ± standard deviation, while categorical variables were presented as percentages and frequencies. Statistical significance was defined as a two-tailed *p*-value ≤ 0.05. Logistic regression was applied to identify the association between dependent and independent variables. Regression analysis was performed in two steps (univariate and multivariate). In the univariate analysis, the association between each dependent and independent variable was assessed and adjusted odd ratios (AORs) and 95% confidence intervals (CIs) were calculated. Multivariate analysis was conducted for all variables showing significance in the univariate analysis (*p*-value ≤ 0.05).

## 3. Results

In this study, we enrolled 277 histopathological proven OC cases and 411 healthy controls.  Table 1 summarizes the statistics of continuous variables, revealing a mean age (51.7 years) of OC onset and no significant difference in BMI between cases and controls.  Table 2 describes the statistics of cases and controls on categorical variables. The majority of OC patients (54.5%) was between 51 and 70 years old, followed by 31–50 years old (40.7%), compared to those under 30 years old. Males accounted for 71.5% of OC cases and had a considerably higher risk than females (28.5%). The majority of OC patients (61.4%) were from the lowest socioeconomic class, mostly from the Punjab province. A significant number of cases were uneducated (58.2%) or had education up to the matric (Grade 10) level.

### 3.1. Descriptive Statistics of Addiction History in Cases and Controls

Table 3 describes the significance of addiction history with the onset of the disease. Cigarette smoking and OC were shown to be significantly correlated in the first descriptive analysis, suggesting a direct relationship. However, this association lost significance in the multivariate regression analysis after adjusting for other confounding variables. Nevertheless, patients with a history of chewing niswar (AOR 6.83; 95% CI: 2.67–17.45, *p*-value: 0.001), areca nut (AOR 4.99; 95% CI: 1.51–16.45, *p*-value = 0.001), and pan (AOR 7.90; 95% CI: 3.19–19.59, *p*-value: 0.001) were identified as significant contributors to OC development. Tea drinking was linked to a significantly greater risk of OC (AOR 4.73; 95% CI: 1.69–13.18, *p*-value: 0.001). Parents from the same clan increased the incidence of OC (AOR 2.15; 95% CI: 1.12–4.14, *p*-value = 0.02). Physical activity appeared to be protective against OC (AOR 0.41; 95% CI: 0.23–0.75, *p*-value: 0.004). While excessive sunlight exposure appeared to be an independent risk factor of OC (AOR 0.15; 95% CI: 0.08–0.28, *p*-value: 0.001). However, huqqa and alcohol were not associated with the development of OC in Pakistan.

### 3.2. Descriptive Statistics on Baseline Tumor-Related Characteristics

Table 4 describes the baseline tumor-related characteristics of the cases. The most common subsites affected in OC were the tongue (40.8%), followed by the buccal mucosa (27.1%), mandible (10.4%), lip (8.3%), and palate (2.9%). Histopathological examination confirmed OC in 90% of cases. The most prevalent histopathological type at diagnosis was moderately differentiated SCC, accounting for 42.6% of cases. Well-differentiated SCC was observed in 38.3%, while poorly differentiated SCC was noted in 6.8% of cases. Regarding tumor staging, pT2 was the most frequently observed (41.3%). Lymph node involvement was absent (N0) in 45.5% of patients, while 54.5% had lymph node metastasis (N1–N3). Among the 169 patients who underwent neck dissection, 28 cases (16.6%) showed extranodal extension (ENE). Additionally, a family history of cancer was observed in 26.7% of cases, with 18.1% having first-degree relatives and 8.3% having second-degree relatives affected by cancer.

### 3.3. Results From Logistic Regression Analysis

Table 5 describes the significant association of independent risk factor with its binary outcome. In the univariate logistic regression model, all 10 variables were recognized as significant independent risk factors for OC. These variables included age, addiction substances like cigarette smoking, niswar, pan, areca, coffee, tea, parents from the same clan, physical activity, and sunlight exposure.

Table 6 describes the significant association between independent risk factors (adjusted values) and dependent factors selected in this study. In the multivariable logistic regression model, six factors appeared as significant independent risk factors for cancer including age, addiction to substances like niswar, pan, areca, tea, and excessive sunlight exposure at 95% CI and *p*=0.001, whereas smoking (AOR 1.12; 95% CI 1.13–1.18, *p*=0.87), coffee consumption (AOR 0.27; 95% CI 0.11–0.66, *p*=0.03), parent same clan (AOR 2.15; 95% CI 1.12–4.14, *p*=0.02), and physical activity (AOR 0.41; 95% CI 0.23–0.75, *p*=0.004) were not identified as associated factors for OC in the multivariate regression model.

## 4. Discussion

OC is a major public health challenge in Pakistan, yet comprehensive data on its incidence, prevalence, and determinants remain insufficient. OC incidence exhibits considerable regional variations influenced by age, gender, geographical location, and demographic patterns. Our study conducted a rigorous analysis to define the determinants of OC within the Pakistani demographic employing robust data collection techniques, including lifestyle evaluations, demographic profiling, and medical histories. Statistical analyses identified significant associations between various risk factors and OC incidence.

Our investigation revealed a strong association between OC development and several demographic and lifestyle factors within the Pakistani population. Notably, increasing age, male gender, low education level, and the consumption of niswar, areca nut, tea, and excessive exposure to sunlight emerged as significant risk factors for OC. However, we found no significant association between OC and the consumption of alcohol or cigarette smoking, which aligns with findings from a study conducted in the Indian population [11]. Conversely, other studies have suggested that the combined use of smoking and alcohol significantly increases OC risk in India [12–14] and Taiwan [15]. These discrepancies could be due to different components and additives in tobacco brands globally or cultural differences in smoking habits. This study underscores the multifactorial nature of OC etiology within the Pakistani context, indicating that the effects of smoking, alcohol, and OC incidence vary across different ethnicities.

Similar to several Southeast Asian countries where it is common to chew raw or processed areca nut alone, in Pakistan, it is commonly consumed alone and also as part of traditional chewing preparations. These include betel quid (also known as paan, which consists of betel leaf, slaked lime, areca nut, and tobacco), gutka (a mixture of areca nut, tobacco, paraffin wax, slaked lime, and flavoring agents), pan masala (containing slaked lime, areca nut, tobacco, and musk ketones), naswar (a blend of tobacco, ash, and lime), as well as other forms such as mainpuri, and mawa (both containing tobacco, lime, and areca nut) and sweet supari [8, 16].

Obesity and higher BMI are recognized as primary causes of patient morbidity and mortality. Although the link between BMI and cancer prognosis is unclear, it is commonly used as an indicator of patient nutritional status in oncological studies. Previous studies have shown that BMI influences treatment outcomes for various cancers [17], with high BMI potentially responsible for approximately 3.6% of all cancer cases [18] and up to 20% according to other estimates [19]. Our results demonstrated a non-significant difference of mean BMI. 25.8 ± 5.8 in OC patients, 25.3 ± 8.6 in controls, indicating that BMI might not be a significant factor of OC in our population. However, in Indian and Chinese populations [20–23], higher BMI is associated with OC, while conflicting evidence from France and Japan associates lower BMI with OC onset and progression [24, 25]. These conflicting results highlight the need for large-scale research to clarify the association between OC and BMI.

Univariate analysis revealed that the age group 51–70 years (54.5%) is significantly associated with OC incidence, consistent with research findings indicating that most OC cases occur in individuals aged 50–70 [26]. Age increases the incidence of OC due to aging-related environmental exposures and physiological changes [27, 28]. Our results validate earlier studies, linking age and OC risk in India [29]. This study also identifies gender difference in OC prevalence, with men showing a higher frequency (71.5%) than women (28.5%), consistent with previous studies in Iran, India, and Pakistan. However, the male-to-female ratio in Thailand [30] indicates a reversed gender ratio. The male preponderance of OC is likely due to environmental and occupational risk factors.

Our findings confirm previous research in Asian nations that low SES and limited education are key drivers of OC [31, 32]. However, low income was not a risk factor for OC in the Turkish population [33]. Our findings also revealed a strong association between OC and the frequent use of areca nuts, pan, and niswar, consistent with studies from Khyber Pakhtunkhwa and Karachi, Pakistan [8].

Cultural practices play a crucial role in shaping oral health outcomes. While thermal injury from hot beverages may contribute to OC by inducing oxidative stress in oral tissues, regional differences in tea preparation, consumption habits, and genetic predisposition may influence cancer risk [34, 35]. Understanding these factors could help clarify the role of tea consumption in OC pathogenesis and aid in developing targeted prevention strategies. Our study identified tea consumption as significant risk factors for OC, aligning with previous findings from Pakistan, Afghanistan, and India [34, 36–38]. Comparable associations have been observed in other populations. For instance, a systematic review of 59 epidemiological studies conducted in Iran examined the potential link between hot tea consumption and esophageal cancer risk [39]. In Los Angeles, California, daily consumption of more than one cup of hot tea has been associated with an increased risk of head and neck cancer (OR, 1.38; 95% CI, 1.09–1.74) [40]. Additionally, a study from India reported a fourfold increased risk of esophageal cancer associated with tea consumption [41]. Conversely, studies conducted in China suggest a potential protective effect of tea against cancer [42–44]. These findings indicate that the impact of tea consumption on cancer risk varies across populations, possibly due to differences in genetic susceptibility, beverage preparation methods, or lifestyle factors. Given the conflicting evidence regarding tea consumption and its role in OC risk, further population-specific research is warranted.

The distribution of OC also varies across anatomical regions and is influenced by ethnic background. Oral habits, particularly those involving the tongue, significantly impact OC development, with variations observed across different countries and regions [45–48]. Our study showed that most common tumor site was tongue and 83.4% of cases were with no ENE, which is clinically important as the absence of ENE is associated with better survival outcomes. While other studies from Pakistan, India, and Taiwan report a higher incidence of OC in the cheek or buccal mucosa [27, 49, 50], highlighting the complex interactions among genetic, environmental, and behavioral factors that contribute to OC development in diverse cultural settings.

Delayed diagnosis and treatment of OC can be complicated by its spread to nearby tissues and lymph nodes [51]. Insufficient education and poor SES likely contribute to advanced OC diagnoses. Previous reports have shown that lower SES influences the disease's course, with a significant percentage of cases detected at an advanced stage [52]. Similar study findings were reported in Malaysia, where some ethnic groups have a high prevalence of late-stage tumors [53], and in Taiwan, where late-stage cancer was common among tobacco users [54]. In Pakistan late-stage cancer diagnoses are often due to poverty, low education, lack of knowledge about cancer symptoms and treatment alternatives, and limited access to affordable medical services. The stigma associated with cancer also deters people from seeking treatment. Comprehensive interventions focused on enhancing healthcare infrastructure, increasing public awareness, and addressing socioeconomic disparities needed to enable earlier detection and treatment.

A key limitation of this study is its reliance on self-reported data, which may introduce recall and social desirability biases. Participants may have underreported their consumption of substances such as alcohol, tobacco, and betel quid due to cultural norms, religious beliefs, or lack of awareness. In Muslim-majority populations like Pakistan, alcohol use is discouraged, which could lead to further underreporting and underestimation of its association with OC. Future studies should incorporate anonymous reporting methods to enhance data accuracy and minimize misclassification biases.

Discrepancies in results may be due to differences in sample size, study design, and population characteristics, emphasizing the need for extensive population-based studies to determine the true association between these risk factors and OC development in Pakistan.

## 5. Conclusion

Our study reveals that environmental factors and consumption habits, such as niswar, pan, areca, and tea, significantly influence OC susceptibility in Pakistani population. Based on an extensive literature review, our study is the first to determine a significant association between such a large number of samples and risk factors with OC onset and development covering all provinces of Pakistan. The study emphasizes the need for public health initiatives to reduce environmental factors affecting OC and improve public health outcomes. It underscores raising public awareness about OC severity, preventing new cases, and improving early diagnosis and treatment options. Further research is needed to confirm these findings and explore other factors influencing OC occurrence in Pakistan. By developing more effective therapies and deepening our understanding of the intricate interactions among lifestyle choices, demographic traits, and OC risk, we can address this urgent public health issue and enhance the well-being of people throughout Pakistan.

## Figures and Tables

**Table 1:** Descriptive statistics for cases versus controls for continuous variables.

Variables categories	Cases (*n* = 277)	Controls (*n* = 411)	*p*-value
Age at OC diagnosis (years)			
mean ± SD^∗^	51.6 ± 10.6	29.7 ± 10.9	< 0.0001
Median (min-max)	52.6 (19.7–77.0)	26 (18–79.5)	
^∗∗^BMI, kg/m^2^			
Mean ± SD	25.9 ± 5.8	25.3 ± 8.6	0.31
Median (min-max)	25.1 (15.6–48.6)	24.46 (15.04–54.5)	

^∗^Standard deviation.

^∗∗^Body mass index.

**Table 2:** Descriptive statistics for cases versus controls for categorical variables.

Variables categories	Cases *n* (%) 277 (40.3%)	Control *n* (%) 411 (59.7%)	*p*-value
Age (categorical)			
18–30	7 (2.5)	255 (62.0)	< 0.0001
31–50	113 (40.7)	130 (31.6)	
51–70	151 (54.5)	25 (6.0)	
Above 70	06 (2.1)	01 (0.2)	
Gender			0.47
Male	198 (71.5)	304 (74.0)	
Female	79 (28.5)	107 (26.0)	
Ethnicity			0.22^a^, 0.46^b^
Punjabi	196 (70.8)	280 (68.1)	
Pathan	54 (19.5)	60 (14.6)	
Kashmiri	04 (1.4)	41 (10.0)	
Balochi	02 (0.7)	15 (3.6)	
Sindhi	10 (3.6)	07 (1.7)	
Mixed/multiracial	09 (3.2)	05 (1.2)	
Afghani	02 (0.7)	03 (0.7)	
Socioeconomic status			< 0.0001
Low	170 (61.4)	94 (22.9)	
Middle	95 (34.3)	315 (76.6)	
High	02 (0.7)	02 (0.5)	
Unknown	10 (3.6)	00 (0.0)	
Education status			
Uneducated	101 (36.5)	27 (6.6)	< 0.0001^c^
Under matric	60 (21.7)	41 (10.0)	
Matric	55 (19.9)	38 (9.2)	
Intermediate	27 (9.7)	38 (9.2)	
Graduates	23 (8.3)	177 (43.0)	
Post graduate	11 (4.0)	90 (21.9)	
Parents relation			
First cousins	102 (36.8)	97 (23.6)	< 0.0001^d^
Second cousins	21 (7.6)	40 (9.7)	
Distant relatives	35 (12.6)	31 (7.5)	
Not related	119 (43.0)	242 (58.9)	
Parents from same clan/zat			0.003
No	123 (44.4)	137 (33.3)	
Yes	154 (55.6)	274 (66.7)	
Other illness			
No	183 (66.1)	297 (72.3)	
Yes	94 (33.9)	114 (27.7)	
Dental disease and treatment	75 (27.1)	79 (19.2)	
Gum disease and treatment	09 (3.2)	08 (1.9)	
Mouth ulcer	03 (2.1)	03 (0.7)	
Conditions for hospitalization other than oral	07 (2.5)	24 (5.8)	
Physical activity			
No	213 (76.9)	205 (49.9)	< 0.0001
Yes	64 (23.1)	206 (50.1)	
Sunlight exposure			< 0.0001
No	138 (49.8)	74 (18.0)	
Yes	139 (50.2)	337 (82.0)	

^a^Punjabi ethnicity versus pathan.

^b^Punjabi ethnicity versus others.

^c^Uneducated versus educated.

^d^Parents with relation/same clan versus with no relation/same clan.

**Table 3:** Descriptive statistics for cases versus controls on addiction history.

Variables categories	Cases 277 (40.3%)	Control 411 (59.7%)	*p*-value
Smoking			0.001
No	176 (63.5)	319 (77.6)	
Yes	101 (36.5)	92 (22.4)	
Huqqa			0.19
No	265 (95.7)	397 (96.6)	
Yes	12 (4.3)	14 (3.4)	
Niswar			0.001
No	193 (69.7)	400 (97.3)	
Yes	84 (30.3)	11 (2.7)	
Pan			0.001
No	202 (72.9)	395 (96.1)	
Yes	75 (27.1)	16 (3.9)	
Areca nut			0.001
No	239 (86.3)	400 (97.2)	
Yes	38 (13.7)	11 (2.6)	
Coffee			0.001
No	255 (92.0)	331 (80.5)	
Yes	22 (7.9)	80 (19.5)	
Tea			0.001
No	09 (3.2)	104 (25.3)	
Yes	268 (96.8)	307 (74.7)	
Alcohol			0.09
No	274 (98.9)	384 (93.4)	
Yes	03 (1.1)	27 (6.6)	

**Table 4:** Clinicopathological characteristics of study cases.

Variables categories	Total 277 (100.0%)
Tumor location	
Tongue	112 (40.4)
Buccal mucosa	74 (26.7)
Mandible	29 (10.5)
Lip	23 (8.3)
Cheek	18 (6.5)
Palate	09 (3.3)
Others^∗^	12 (4.3)
Histological type	
Poorly differentiated SCC	19 (6.8)
Moderately differentiated SCC	118 (42.6)
Moderate to poorly differentiated SCC	05 (1.8)
Well differentiated SCC	106 (38.3)
Well to moderately differentiated SCC	02 (0.7)
SCC	24 (8.7)
Verrucous SCC	02 (0.7)
Basaloid SCC	01 (0.4)
Pathological T	
pT	03 (1.6)
pT1	38 (20.6)
pT2	76 (41.3)
pT3	36 (19.6)
pT4	29 (15.8)
pTis	02 (1.1)
Not known	93
Pathological N	
N0	70 (45.5)
N1	35 (22.7)
N2	33 (21.4)
N3	16 (10.4)
Not known	123
Extranodal extension (ENE) status	
Present	28 (16.6)
Absent	141 (83.4)
No neck dissection	108
Pathological M	
pMx	277
Family history of cancer	
No	203 (73.3)
Yes	74 (26.7)
1^st^ degree relatives	
No	227 (81.9)
Yes	50 (18.1)
2^nd^ degree relatives	
No	254 (91.7)
Yes	23 (8.3)

^∗^Others = maxilla (*n* = 4), left/right commissure (*n* = 3), jaw (*n* = 2), supraglottic region (*n* = 1), left retromolar area (*n* = 1), left upper posterior alveolus (*n* = 1).

**Table 5:** Univariate logistic regression of sociodemographic and potential risk factors associated with OC.

Variable categories	^∗∗^UOR	95% ^∗∗∗^CI	*p*-value
Age			
Mean ± ^∗^SD	1.59	(1.13–1.18)	0.001
Smoking			
No	Ref.	Ref.	
Yes	2.03	(1.41–2.91)	0.001
Niswar			
No	Ref.	Ref.	
Yes	15.8	(8.25–30.36)	0.001
Pan			
No	Ref.	Ref.	
Yes	9.20	(5.20–16.14)	0.001
Areca nut			
No	Ref.	Ref.	
Yes	5.30	(2.71–10.32)	0.001
Coffee			
No	Ref.	Ref.	
Yes	0.36	(0.22–0.59)	0.001
Tea			
No	Ref.	Ref.	
Yes	12.73	(5.82–27.85)	0.001
Parents from same clan/zat			
No	Ref.	Ref.	
Yes	1.87	(1.30–2.66)	0.001
Physical activity			
No	Ref.	Ref.	.
Yes	0.29	(0.21–0.42)	0.001
Sunlight exposure			
No	Ref.	Ref.	
Yes	0.22	(0.16–0.31)	0.001

^∗^Standard deviation.

^∗∗^Unadjusted odd ratio.

^∗∗∗^Confidence interval.

**Table 6:** Multivariate logistic regression of sociodemographic and potential risk factors associated with OC.

Variables categories	^∗∗^AOR	95% ^∗∗∗^CI	*p*-value
Age			
Mean ± ^∗^SD	1.12	(1.13–1.18)	0.001
Smoking			
No	Ref.	Ref.	
Yes	0.95	(0.51–1.77)	0.87
Niswar			
No	Ref.	Ref.	
Yes	6.83	(2.67–17.45)	0.001
Pan			
No	Ref.	Ref.	
Yes	7.90	(3.19–19.59)	0.001
Areca nut			
No	Ref.	Ref.	
Yes	4.99	(1.51–16.45)	0.001
Coffee			
No	Ref.	Ref.	
Yes	0.27	(0.11–0.66)	0.03
Tea			
No	Ref.	Ref.	
Yes	4.73	(1.69–13.18)	0.001
Parents from same clan/zat			
No	Ref.	Ref.	
Yes	2.15	(1.12–4.14)	0.02
Physical activity			
No	Ref.	Ref.	
Yes	0.41	(0.23–0.75)	0.004
Sunlight exposure			
No	Ref.	Ref.	
Yes	0.15	(0.08–0.28)	0.001

^∗^Standard deviation.

^∗∗^Adjusted odd ratio.

^∗∗∗^Confidence interval.

## Data Availability

Data sharing is not applicable to this article as no new data were created or analyzed in this study.
